# A164 IDENTIFYING THE MOST IMPORTANT PREDICTORS TO CORRELATE SERUM METABOLITES WITH MRE CHANGES IN PATIENTS WITH PEDIATRIC CROHN DISEASE

**DOI:** 10.1093/jcag/gwac036.164

**Published:** 2023-03-07

**Authors:** R G Suarez, N Guruprasad, G Tata, Z Zhang, G Focht, V Navas-López, S Koletzko, A M Griffiths, D Wishart, E Wine

**Affiliations:** 1 Pediatrics; 2 Computing Science, University of Alberta, Edmonton, Canada; 3 Food Nutrition and Gastrointestinal Microbiome, Jimei University, Xiamen, China; 4 Hebrew University, Jerusalem, Israel; 5 HRU, Malaga, Spain; 6 LMU, Munich, Germany; 7 Hospital for Sick Children, Toronto, Canada

## Abstract

**Background:**

Endoscopy has been the gold standard for assessing activity in Pediatric Crohn disease (pCD); however, it is limited by its invasiveness and partial assessment of small intestine and transmural inflammation. To that end, the Pediatric Inflammatory Crohn's MRE Index (PICMI) is a valid, reliable, non-invasive, and responsive index that includes transmural inflammation when assessing disease activity.

The pathogenesis of pCD remains poorly understood, but evidence suggests that endogenous metabolites produced in the intestinal tract might mediate pathogenesis. Despite the important applicability of metabolomics in increasing the understanding of pCD, there has been limited research on this topic.

**Purpose:**

Serum metabolomic profiles are linked to disease activity in pediatric Crohn disease.

**Method:**

ImageKids is a multicenter, prospective, observational cohort study, designed to develop PICMI for pCD. The study was conducted over 18 months with paired serum specimens collected at study initiation and completion for 56 pCD patients. Due to the long time between the visits and the fact that during the study variables that highly affect serum metabolites were not controlled, we considered each patient visit as an individual measure point. Metabolites were identified using a quantitative metabolomics approach through The Metabolomics Innovation Centre (TMIC; University of Alberta). Disease activity was determined by the cutoff values in the total PICMI score of each patient.

The most relevant serum metabolites were identified by medium-level and high-level variable selection analysis. Pearson correlation and hypothesis testing were used to select important metabolites. Decision trees, regularization techniques, and support vector machines were used to assess explicit importance of metabolites in disease activity.

**Result(s):**

This work provides a strategy to reduce a dimensional dataset from a metabolomic experiment. By medium-level selection analysis we were able to identify 117 statistical important metabolites for disease activity. The high-level selection analysis allowed to indicate the importance of the top 10 metabolites trough disease activity (defined by PICMI index). Results, also show that the evaluation of importance of metabolites through multivariate statistical models is dependent of the intrinsic variable selection model.

Figure 1 reveals that Tryptophan ranked highest in the feature importance scoring. Histidine, Methylhistidine, Citric acid, Isoleucine, and Decanoylcarnitine also correlated well with disease severity.

**Image:**

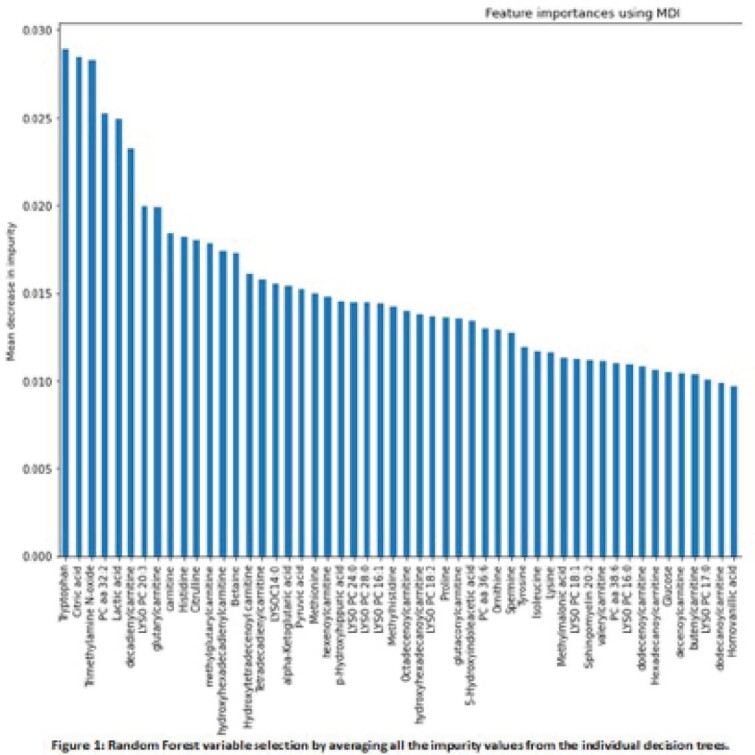

**Conclusion(s):**

This work uses a unique approach of multivariate statistical analyses, to identify metabolites associated with pCD disease activity. Tryptophan has been previously identified as significantly altered in the blood of IBD patients compared to controls. Histidine is known to be involved in the mediation of oxidative stress, potentially influencing intestinal inflammation. These metabolites could serve as biomarkers and help define pCD pathogenesis.

**Disclosure of Interest:**

None Declared

